# CMV IgG in the blood is not associated with hepatitis but correlates with poor outcomes in immunotherapy treated melanoma patients

**DOI:** 10.1007/s00262-024-03859-3

**Published:** 2025-01-03

**Authors:** Sophia B. Strobel, Devayani Machiraju, Melanie Wiecken, Jasmin Richter, Julian A. F. Klein, Annemarie Berger, Jessica C. Hassel

**Affiliations:** 1 Department of Dermatology and National Center for Tumor Diseases (NCT), Medical Faculty Heidelberg, NCT Heidelberg, a partnership between DKFZ and University Hospital Heidelberg, Heidelberg University, Heidelberg, Germany; 2 Faculty of Biosciences, Heidelberg University, Heidelberg, Germany; 3 Department of Dermatology, Venereology and Allergology, University Medical Center, Medical Faculty Mannheim, University of Heidelberg, Mannheim, Germany; 4 Department of Virology, Centre for Infectious Diseases, University Hospital Heidelberg, Heidelberg, Germany; 5 University Hospital, Institute for Medical Virology, Goethe University Frankfurt, Frankfurt, Germany

**Keywords:** Melanoma, CMV, IgG, IgM, Hepatitis, Immunotherapy, Survival, Peripheral Immune cells

## Abstract

Cytomegalovirus (CMV) infection or reactivation in immune-compromised individuals can lead to a wide range of severe complications including hepatitis. However, its relation with immune checkpoint inhibitors (ICIs) induced hepatitis (ICI-hepatitis) and tumor responses in advanced melanoma patients remains unclear. Hundred and ninety metastatic cutaneous melanoma patients (mCM) who received ICI treatment, with CMV IgG or IgM information available at baseline, were included in the study (Cohort 1). Clinical characteristics and immune cell count in the blood were retrieved from medical records. In addition, anti-CMV IgG and IgM were measured in pre and on-treatment serum samples from 49 advanced skin cancer patients using ELISA (Cohort 2). In the event of a positive anti-CMV IgM, further analysis with PCR was performed. Univariate and multivariate analysis was used to assess the relationship between CMV IgG or IgM and ICI-hepatitis tumor outcomes. Twenty-one patients (11%) developed hepatitis during ICI treatment (Cohort 1). ICI-hepatitis was significantly associated with disease control rate (DCR; *p* = 0.017) and longer progression-free survival (PFS; *p* = 0.008) in mCM patients. Detection of CMV IgG or IgM antibodies were not associated with ICI-hepatitis (*p* > 0.05). However, increased CMV IgG values at baseline correlated with disease progression (*p* = 0.047) and shorter PFS (*p* = 0.081). In addition, increased CMV IgG values were associated with reduced levels of monocytes (*p* = 0.005), eosinophils (*p* = 0.062), and neutrophils (*p* = 0.065) in the blood. In summary, anti-CMV IgG or IgM in the blood may not be associated with ICI-hepatitis, but high anti-CMV IgG at baseline indicates poor outcomes in ICI-treated mCM patients.

**Supplementary Information:**

The online version contains supplementary material available at 10.1007/s00262-024-03859-3.

## Introduction

Immune checkpoint inhibitors (ICIs) against cytotoxic T‐lymphocyte‐associated antigen 4 (CTLA‐4) and programmed cell death 1 (PD‐1) reactivate immune cells and have shown promising anti-tumor responses in a subset of advanced melanoma patients [1]. However, the immune activation induced by these therapies can often lead to off-target toxicities that resemble autoimmune diseases [2]. ICI-induced immune‐related adverse events (ir-AEs) most often affect the skin, gastrointestinal tract, liver, endocrine organs, and lungs [3]. Among them, ICI-induced hepatitis is reported to occur in 1–29% of patients on treatment [4, 5]. The incidence varies with the ICI drug class and appears synergistic in combination therapy targeting CTLA‐4 and PD‐1. Here, up to 17% of patients reveal a high‐grade toxicity of grades 3 and 4, that needs to be treated with immunosuppressive therapy [5].

Hepatitis is due to liver inflammation and can be caused by several factors, including viral infections, medications, or metabolic diseases [6]. Cytomegalovirus (CMV) is a common herpes virus, and once infected, the human body retains the virus for life. Therefore, it rarely causes problems in healthy people [7]. However, despite the absence of overt symptoms, there is evidence that infected individuals may have long-term adverse outcomes related to the induction of a chronic inflammatory cell-mediated immune response to this innocuous virus [8]. In addition, it is essential to note that the reactivation of CMV in a few people with a weakened immune system may lead to signs and symptoms, including severe organ-specific complications such as hepatitis and colitis [7, 9]. CMV can infect most liver cells, including hepatocytes and hepatic endothelial cells [10–12]. In hepatocytes, CMV has shown to exert a direct cytopathic effect, and in hepatic endothelial cells, CMV infection facilitates recruitment and activation of distinct functional CD4 T cell subsets driving liver inflammation. Besides, it is the most common viral infection in recipients of liver transplantation, and compared to primary infection, reactivation of CMV infection is more common in these patients. Meanwhile, a weakened immune system is one of the hallmarks of cancer, suggesting that CMV infection or reactivation in these patients may be capable of driving hepatitis. However, hepatitis was also observed in a subset of melanoma patients receiving ICIs [13]. Although the mechanisms behind the pathogenesis are yet to be explored, the immune checkpoint proteins are known to be involved in maintaining the tolerogenic environment in the liver, and unblocking these proteins by ICI therapy may drive liver inflammation. Interestingly, as this was only observed in a subset of patients, some predisposing factors may account for the incidence. In this context, given the potential of CMV infection or reactivation to drive hepatitis, it is important to know the influence of CMV infection/reactivation on ICI-induced hepatitis.

Moreover, the clinical relevance of CMV in cancer patients remains a matter of debate. On one side, CMV was suggested as a potential therapeutic target in cancer patients due to its contribution to the development of tumorigenesis [14, 15]. In vitro, CMV infection of already transformed malignant cells have shown to favor the development of tumor growth [16]. In addition, persistently human CMV-infected neuroblastoma cells resulted in increased malignant behavior and treatment resistance [17, 18]. In contrast, treatment of B16-F10 melanoma xenografts with murine CMV-derived MHC peptide epitopes caused an increase in cancer-protective immune cells and, and reduced tumor volume [19], suggesting the role of CMV on anti-tumor immune responses. In addition, there no systematic analysis to understand the influence of CMV on ICI responses in melanoma patients.

Besides, due to the mild symptoms in immunocompetent patients and many mild to moderate liver elevations during therapy, the impact of CMV reactivation in the context of checkpoint inhibitor therapy remains uncertain. Hence, in this study, we investigated the association of anti-CMV IgM and IgG values in the blood of advanced melanoma patients with ICI-induced hepatitis and clinical outcome.

## Materials and methods

### Patients and samples

#### Cohort 1

Metastatic cutaneous melanoma patients (mCM) receiving ICIs, either anti-PD-1 antibody alone (pembrolizumab or nivolumab) or a combination of anti-CTLA4 and anti-PD-1 antibody (ipilimumab + nivolumab), with information available on anti-CMV IgG or IgM serostatus before ICI treatment between March 2013 and December 2021 were included in this retrospective analysis. In this cohort, only the routinely documented information on CMV IgG and IgM from the clinic was considered for the analysis. Pre-treatment values of anti-CMV IgM and IgG, the absolute number of immune cells including lymphocytes, monocytes, neutrophils, eosinophils, and basophils in the blood, and standard clinical features such as age, gender, LDH and prior systemic treatments were retrieved from the medical records. The Ethical Committee of the Medical Faculty of Heidelberg approved the retrospective analysis of patient data and biobanking of blood samples (S-454/2015, S-207/2005).

##### CMV information in the cohort 1

In this cohort, CMV IgG and IgM antibody testing were conducted with Enzygnost® Anti-CMV/IgG and Enzygnost® Anti-CMV/IgM, using the ELISA Processor BEP® III System (Siemens Healthcare, Erlangen, Germany). In order to verify active CMV infection or reactivation, positive IgM-ELISA results were further investigated, either via polymerase chain reaction (PCR) or pp65 antigen immunofluorescence test (IFT) from blood. We conducted CMV-PCR from blood plasma or serum, DNA extraction was performed with QIAsymphony sp instrument (Qiagen®, Hilden, Germany). RealStar® CMV PCR Kit 1.0 (altona Diagnostics®, Hamburg, Germany) and Roche LightCycler® 480 Instrument (Roche Diagnostics, Mannheim, Germany) were utilized for CMV-PCR. The CMV pp65 Antigenemia Antibody Set by Millipore Light Diagnostics™ (Temecula, United States of America) was used to detect the pp65 antigen via IFT.

#### Cohort 2

In cohort 2, patients from whom frozen serum samples were available in the biobank to look at CMV sequential results during the ICI treatment were included prospectively. Pre and on-treatment frozen serum samples were available from 49 patients with advanced skin cancer receiving ICIs. Among them, 22 patients (45%) overlap with cohort 1. Anti-CMV IgG and IgM values from frozen serum samples were measured at three time points (before, after 6 weeks, and after 12 weeks of ICI treatment). In the event of a positive anti-CMV IgM, PCR was performed on all of these samples.

##### CMV information in cohort 2

*Serum collection* Peripheral blood samples were collected and processed according to the standard NCT biobank protocols. Written informed consent was obtained from the patients. The blood samples were centrifuged at 2,500 × g for 10 min for serum separation, divided into 200–300uL aliquots, and stored at –80 °C until further analysis.

*CMV-DNA detection* After DNA extraction using the DSP Virus/Pathogen Midi Kit on the QIAsymphony sp instrument (Qiagen, Hilden, Germany) CMV-specific DNA was detected using an internally controlled real time PCR assay as previously described [20].

*Serological assays* Anti-CMV IgG and IgM antibody screening was performed with the LIAISON® CMV IgG and IgM assays (DiaSorin, Dietzenbach, Germany) according to the manufacturer’s instructions.

### Statistical analysis

All statistical analyses were done using SPSS version 29 (IBM, Ehningen, Germany). The relation between clinical parameters and CMV serology titers was evaluated by the Chi-square or MWU test. The median of anti-CMV IgG titers in cohort 1 was 1:19,000, and for statistical evaluations, all values less than or equal to 1:19,000 were grouped as low, and values above 1:19,000 were grouped as high. Survival curves were generated by the Kaplan–Meier and compared using the log-rank test. Univariable and multivariable analyses were conducted with regression models. Variables with a p value < 0.1 in univariable analysis were tested in multivariable analysis. Progression-free survival (PFS) was defined as the time from the start of ICI until disease progression; when not progressed, patients were censored at the last contact date. Overall survival (OS) was defined as the time from the start of ICI until death, and patients who were alive were censored at the date of the last contact. The date cutoff for OS follow-up analysis in this study was 15th Feb 2023. The bar graphs for data visualization were created using GraphPad Prism version 8 (GraphPad Software, Inc., La Jolla, CA, USA). The bars and lines in the column graphs represent median values and 95% CI. *p* < 0.05 was considered to indicate a statistically significant difference.

## Results

### Patient characteristics (Cohort 1)

In this retrospective analysis, one hundred and ninety patients with stage IV cutaneous melanoma who received ICIs at the Section of Dermato-Oncology, Department of Dermatology, and NCT Heidelberg were included. 55% received anti-PD-1 monotherapy (pembrolizumab (P) or nivolumab (N)), and 45% received a combination of nivolumab and ipilimumab (N + I). Approximately 61% of all patients received ICIs as first-line systemic treatment, and 26% of patients had liver metastasis at the beginning of ICI treatment. The median age was 64 years (range: 20–94), and 57% of the patients were men. The disease control rate (DCR) was 62%, the median PFS was 6.5 (95% CI: 5–9) months, and the median OS was 27 (95% CI: 19–30) months. Immune-related adverse events of any grade were observed in 50% of patients, and hepatitis was observed in 11% of patients. Hepatitis was diagnosed at a median of 9 (95% CI: 5–12) weeks after treatment initiation and more frequent in patients receiving combination immunotherapy with N + I (Table 1). Moreover, 43% of patients who developed hepatitis on treatment had also experienced other irAEs such as thyroiditis, hypophysitis, colitis, etc.

**Table 1:** Patient Characteristics split based on ICI-induced hepatitis in cohort 1

	Hepatitis (*n* = 21)	No Hepatitis (*n* = 169)		*p*-value
Age in years (range)	54 (28–74)	65 (20–94)		0.095
*Gender (n (%))*				1
Male	12 (57)	96 (57)		
Female	9 (43)	73 (43)		
*Prior systemic treatment (n (%))*				0.057
Yes	4 (19)	71 (42)		
No	17 (81)	98 (58)		
*Liver metastases (n (%))*				0.600
Yes	7 (33)	43 (25)		
No	14 (67)	126 (75)		
*Type of ICI (n (%))*				< 0.001
Pembro/Nivo	4 (19)	100 (59)		
Ipi + Nivo	17 (81)	69 (41)		
*LDH (n (%))*				1
Normal	16 (76)	122 (72)		
Elevated	5 (24)	46 (27)		
Missing		1 (< 1)		
*irAEs (n (%))*				< 0.001
Yes	21 (100)	74 (44)		
No	0 (0)	95 (56)		
*Response (n (%))*				0.017
PD	3 (14)	70 (41)		
DCR	18 (86)	99 (59)		
PFS in months (median (95% CI))	26 (6–71)	6 (4–9)		0.011
OS in months (median (95% CI))	30 (26–73)	25 (17–30)		0.073

### ICI-induced hepatitis is associated with better ICI treatment outcomes

**Fig. 1:**
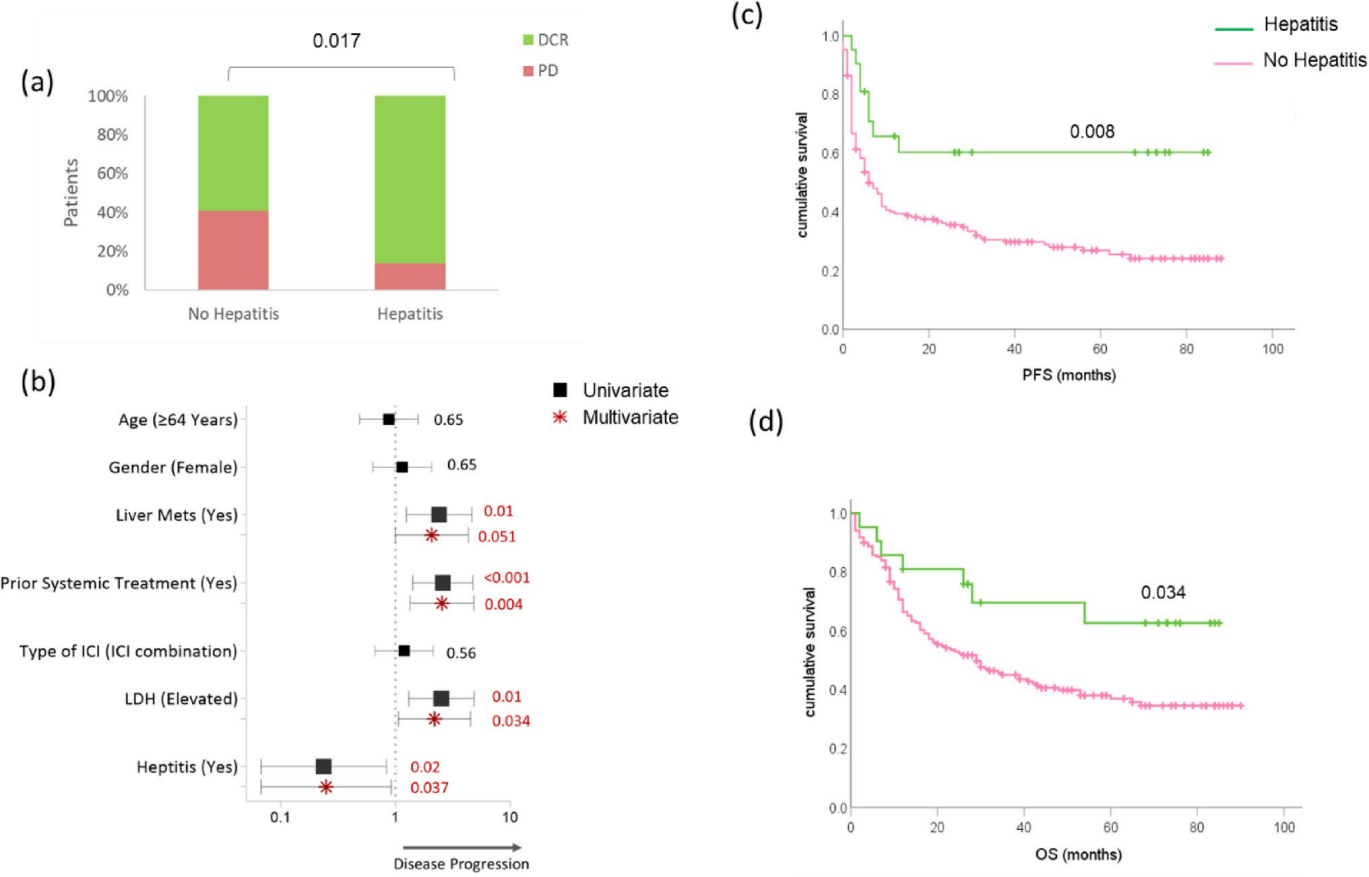
ICI-induced hepatitis was associated with better tumor control and survival on ICI treatment in mCM patients: **a** The bar graph indicates the percentage of patients with or without ICI-induced hepatitis sorted according to the treatment response. The red color represents progressive disease (PD), and the green color represents disease control (DCR). *p*-values are indicated above the group. **b** Forest plot representing the clinical factors associated with tumor response in uni (black) and multivariate (red) analysis. The symbols indicate the hazard ratios (HR), and lines indicate the lower and upper 95% CI. *p*-values are mentioned next to the lines of respective clinical factor. The red color indicates a significant association of a factor with tumor response. **c**, **d** Kaplan Meier curves for survival according to hepatitis (green) or no hepatitis (pink), (**c**) PFS, and **d** OS. *p*-values refer to the log-rank test

Twenty-one patients (11%) developed hepatitis after initiating ICI treatment (19% monotherapy, 81% combination therapy). We observed that patients who developed hepatitis on ICI treatment were less likely to experience disease progression compared to the patients who did not develop hepatitis (Fig. 1a, b *p* < 0.05). Accordingly, a better PFS (*p* < 0.05; HR: 0.407; 95%CI: 0.199–0.833) and OS (*p* < 0.05; HR: 0.450; 95%CI: 0.209–0.96) in patients who developed hepatitis compared to patients who did not develop hepatitis was noted (Fig. 1c, d). Besides, ICI-hepatitis remained a significant factor associated with disease control response (*p* = 0.037; HR: 0.2; 95%CI: 0.1–0.9; Fig. 1b) when adjusted for other potential variables such as liver metastasis (*p* = 0.051; HR: 2.1; 95%CI: 1.0–4.3), prior systemic treatments (*p* = 0.004; HR: 2.5; 95%CI: 1.3–4.8), and LDH (*p* = 0.034; HR: 2.2; 95%CI: 1.1–4.5). We did not observe any differences in responses between patients receiving ICI combination therapy and monotherapy in our study (*p* = 0.56; HR: 1.2; 95%CI: 0.6–2.1). Interestingly, despite more patients receiving ICI combination therapy experiencing hepatitis (81%), we did not observe any influence with the type of ICI regime on the relationship between hepatitis and response. However, it has to be noted that only four patients (19% had developed heptatis during PD1 monotherapy. Meanwhile, the same trend could be seen between hepatitis and PFS (*p* = 0.017; HR: 0.42; 95%CI: 0.2–0.86) in multivariate analysis (Supplementary Fig. 1).

### ICI-induced hepatitis is not associated with CMV reactivation

**Fig. 2:**
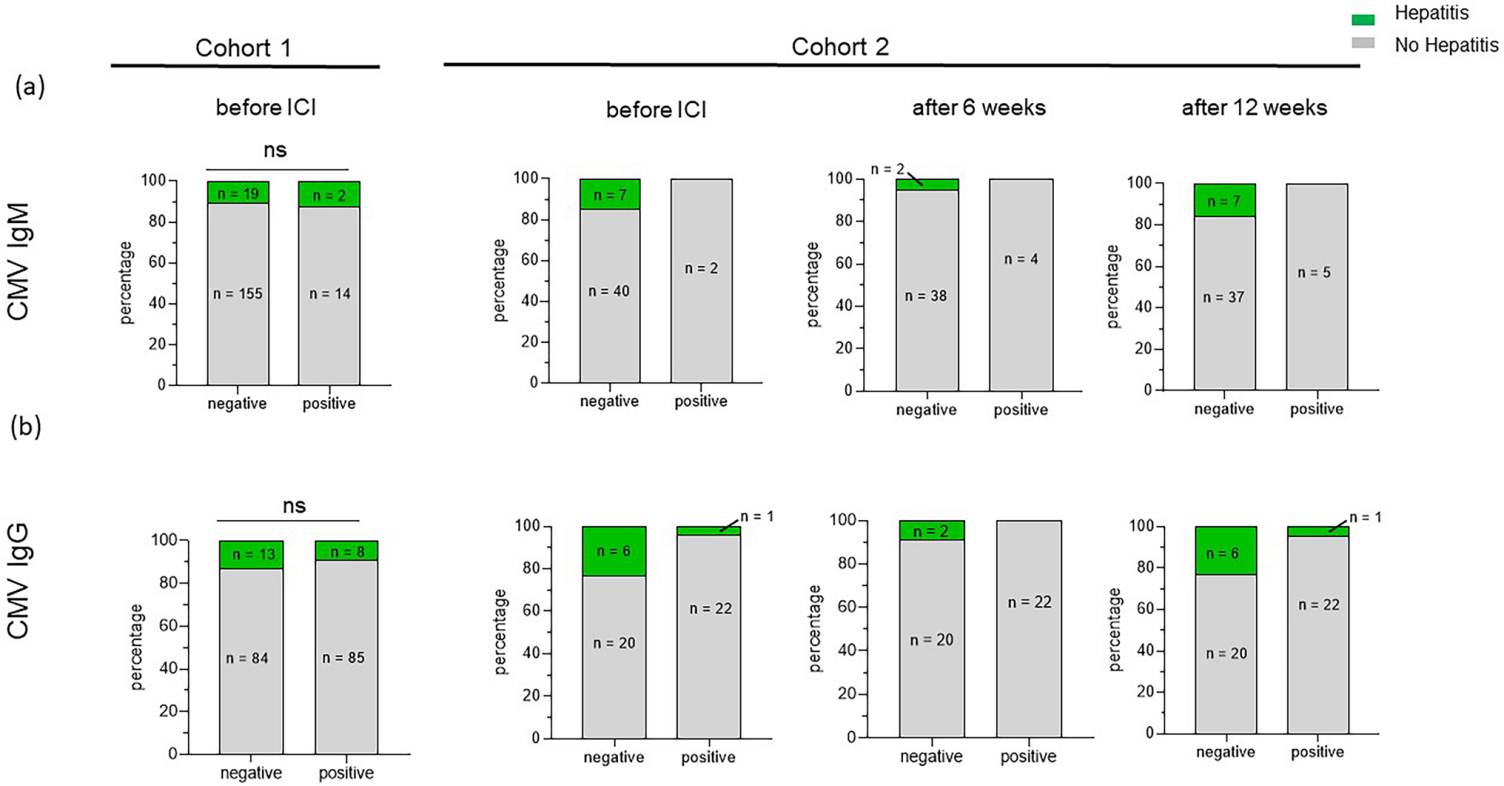
CMV IgM or IgG was not associated with ICI-induced hepatitis. (**a**) Number of hepatitis cases (bars in green) versus patients with no hepatitis (bars in gray) in CMV IgM (**a**) and IgG (**b**) positive and negative patients. No significant difference (ns) was found at baseline in Cohort 1, however, in Cohort 2 the numbers of CMV positive patients or/and hepatitis cases was too low to perform statistics

In cohort 1 of hundred and ninety melanoma patients, 16 patients (9%) were positive for CMV IgM before the initiation of ICI treatment (Fig. 2a). Ninety-three patients (49%) were positive for CMV IgG with a median IgG ratio of 1:19,000, among them 42 patients were considered CMV IgG high based on cutoff at 1:19,000 (Fig. 2b). In 14 (88%) of the CMV IgM positive patients, further validation via pp65 antigen assay or PCR was performed. In only one of these patients an acute infection could be found via positive pp65 antigen IFT result. In 5 of 16 CMV IgM positive patients (31%), follow-up anti-CMV-IgM results were available for on-treatment, and all patients with positive anti-CMV IgM results remained positive during a follow-up time of 7 months (range 4–10 months).

Meanwhile, among the 21 patients who developed hepatitis in cohort 1, only two had positive anti-CMV IgM at baseline (Fig. 2a). Besides, of these 21 patients, 10 patients had follow-up CMV antibodies analyzed when liver values increased. Among the 10 patients, 2 patients with positive anti-CMV IgM at baseline remained positive, and five remained negative during the treatment. However, one patient turned positive for IgM, and two patients had borderline anti-CMV IgM values during the treatment. All those patients with positive or borderline anti-CMV IgM were further evaluated with PCR and found to be negative. Meanwhile, 13 out of 21 hepatitis patients were negative for anti-CMV IgG at baseline; whereas, eight patients were positive for anti-CMV IgG with a median ratio of 1:18,000 (range: 1:3500–1:26,000). Upon applying the IgG cutoff of 1:19,000, seventeen of the hepatitis patients were considered as low/negative and four patients were considered as CMV IgG high (Fig. 2b). Besides, the follow-up information on CMV IgG titers at the onset of hepatitis in these patients revealed an increase in CMV IgG titers by a median of 1:9000 (range: 2000–32000).

To understand the relevance of on-treatment CMV titers to ICI-hepatitis, we used a second cohort of 49 patients and analyzed serum anti-CMV IgG and IgM at three different time points (before, 6 weeks, and 12 weeks after ICI initiation; Fig. 2; cohort 2)). This cohort consisted of ICI-treated patients with different skin cancer diagnoses, majorly cutaneous melanoma (*n* = 39, 80%), followed by uveal melanoma (*n* = 6, 12%), basal cell carcinoma (*n* = 2, 4%), squamous cell as well as Merkel cell carcinoma (*n* = 1, 2% each; Suppl. Table. 1). Based on the clinical diagnosis, 25 patients (51%) received anti-PD1 monotherapy and 24 patients (49%) combination immunotherapy. In this cohort, seven patients developed hepatitis (14%); among them, 6 (86%) patients received ICI combination therapy. Similar to cohort 1, we observed that the development of hepatitis was associated with disease control (*p* = 0.08) but not with PFS and OS, keeping in mind that different cancer types were included here.

Out of forty-nine patients, 23 (47%) had a positive anti-CMV IgG status with consistent values at all three time points measured (one data point is missing at week 6). The remaining patients had negative CMV IgG results throughout treatment. Meanwhile, only two patients (4%) had positive CMV IgM before ICI and remained positive throughout ICI treatment. Two patients who were negative for CMV IgM before ICI were CMV IgM positive at week 6, but only one of them remained positive until week 12. However, we observed the development of positive CMV IgM in two patients only at the last time point, at 12 weeks of ICI (Fig. 2a). PCR was performed to determine CMV viral load in the patients with positive CMV IgM; however, no CMV DNA was detected in any of these patients. Similar to the findings from cohort 1, we did not observe any association between ICI-hepatitis and CMV IgG or IgM at baseline or during the treatment in cohort 2 (Fig. 2; cohort 2). Furthermore, in cohort 2, among seven patients who developed hepatitis, all patients were CMV naive (CMV IgG and IgM negative) except for one patient who had a high CMV IgG (> 250 U/ml) at baseline and stayed high (> 250 U/ml) at the onset of hepatitis.

### Increased CMV IgG titers in the pre-treatment blood samples are associated with progressive disease and reduced immune cells in the blood

Next, we analyzed the relation between CMV IgM and IgG with treatment response and survival in ICI-treated mCM patients(cohort 1, *n* = 190; Table 2).

**Table 2:** Patient characteristics split based on median (≤ 1:19,000) CMV IgG titers in cohort 1

	low CMV IgG (*n* = 148)	high CMV IgG (*n* = 42)	*p*-value
Age in years (range)	63 (20–90)	64 (40–94)	0.212
*Gender (n (%))*			0.112
Male	89 (60)	19 (45)	
Female	59 (40)	23 (55)	
*Type of ICI (n (%))*			0.380
Pembro/Nivo	78 (53)	26 (62)	
Ipi + Nivo	70 (47)	16 (38)	
*LDH (n (%))*			0.843
Normal	107 (72)	31 (74)	
Elevated	41 (28)	10 (24)	
Missing		1 (2)	
*Hepatitis (n (%))*			1.00
Yes	17 (11)	4 (10)	
No	131 (89)	38 (90)	
*Other irAEs (n (%))*			0.054
Yes	79 (53)	15 (36)	
No	69 (47)	27 (64)	
*CMV IgM (n(%))*			< 0.001
Positive	5 (3)	11 (26)	
Negative	133 (90)	28 (67)	
Borderline	10 (7)	3 (7)	
*Response (n (%))*			0.047
PD	51 (34)	22 (52)	
DCR	97 (66)	20 (48)	
PFS in months (median (95% CI))	8 (6–15)	3 (2–9)	0.113
OS in months (median (95% CI))	29 (22–35)	14 (9–29)	0.063

**Fig. 3:**
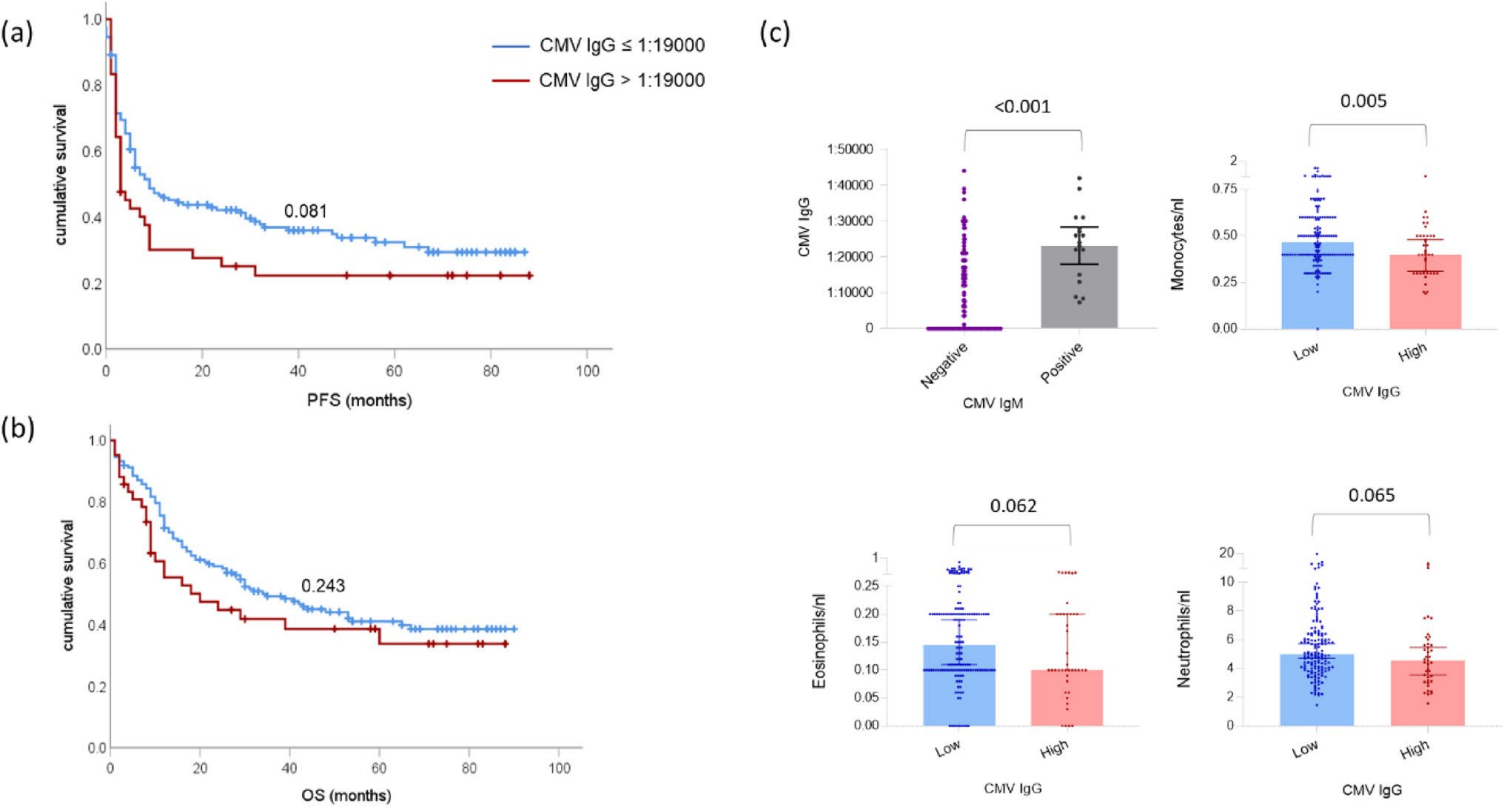
Increased CMV IgG titers correlate with PFS, and a reduced number of monocytes in the peripheral blood: Kaplan–Meier curves for survival according to CMV IgG ≤ 1:19,000 (blue) or > 1:19,000 (red), (**a**) PFS (**b**) OS. *p*-values refer to the log-rank test. (**c**) Bar charts showing the association between CMV IgG and CMV IgM (top, left). The correlation between CMV IgG titers and peripheral absolute immune cell counts: monocytes (*p* = 0.005; top, right), eosinophils (*p* = 0.062; bottom, left), and neutrophils (*p* = 0.065; bottom, right). The lines in the graphs represent the median and 95% CI, respectively. *p*-values are presented above the respective group on top of the chart

Although we found no correlation between CMV IgM or IgG levels in the blood with ICI-induced hepatitis, we found a significant association between CMV IgG titers and ICI treatment response. Patients with high CMV IgG titers at pre-treatment were more likely to experience disease progression on ICI treatment (*p* = 0.047). In addition, CMV IgG titers remained a potential variable associated with disease control response (*p* = 0.063; HR: 2.08; 95%CI: 0.96–4.50) when adjusted for potential clinical variables associated with response from univariate analysis such as LDH, line of systemic treatment, liver metastases, hepatitis as described earlier in the results Sect. “ICI-induced hepatitis is associated with better ICI treatment outcomes” (Supplemental Fig. 2a). Accordingly, a trend for reduced PFS in patients with high CMV IgG titers was observed (*p* = 0.081; Fig. 3a). However, when analyzing ICI regimes individually (supplemental Fig. 2b), we found that CMV IgG relation with disease control response is more prominent in patients who received Ipi + Nivo combination therapy (*p* = 0.05; HR: 3.0; 95%CI: 0.97–9.23) compared to PD1 monotherapy (*p* = 0.24; HR: 1.7; 95%CI: 0.69–4.23; Supplemental Fig. 2b). Moreover, concerning the CMV IgG cutoff, the detection of CMV IgG antibodies itself correlated significantly with disease progression on Ipi + Nivo combination therapy (*p* = 0.03; HR: 2.7; 95%CI: 1.1–6.5). Interestingly, apart from 2 patients, all patients with high CMV IgG ratio were positive for CMV IgM with a significant correlation between both values (*p* < 0.001, Fig. 3c). Nevertheless, we did not observe any relation between CMV IgM and response, PFS, or OS (*p* > 0.1; Supplementary Fig. 3a, b). We also did not observe any difference in CMV IgM even after pooling the two cohorts, and considering CMV IgM positive during ICI therapy (*p* = 0.343; Supplemental Fig. 3c).

In addition to treatment outcomes, we compared CMV IgG levels with the different immune cell subsets in the peripheral blood and found an inverse relationship between high CMV IgG titers and peripheral immune cell counts. High CMV IgG titers significantly correlated with a reduced number of monocytes (*p* = 0.005) and were likely to have less number of eosinophils (*p* = 0.062) and neutrophils (*p* = 0.065) in the blood (Fig. 3c). Meanwhile, no correlation between CMV IgG titres and lymphocytes was observed in our study (Supplemental Fig. 3d). Although analyzing the CD4/CD8 polarization would be of interest, this could not be demonstrated in this study as the CD4/8 ratio is not done in clinical routine and we do not have biobank material to be able to investigate this retrospectively.

## Discussion

This study examined the relationship between markers of CMV infection, hepatitis incidence, and tumor responses in ICI-treated advanced melanoma patients. We observed that patients who developed hepatitis upon ICI treatment significantly experienced better disease control responses and longer PFS. However, we did not observe any correlation between CMV IgG or IgM levels in the blood and ICI-induced hepatitis. Interestingly, we found that patients with high CMV IgG titers before ICI treatment are more likely to experience disease progression and shorter PFS on ICI treatment. In line with this finding, a negative correlation between CMV IgG levels and absolute monocytes, eosinophils, and neutrophils in the peripheral blood was noted.

The liver has tolerogenic mechanisms in place to prevent aberrant immune activation. CTLA4 and PD1 pathways are essential for tolerance induction in this organ [21–23]. PDL1 expression by hepatocytes and non-parenchymal cells helps induce T cell apoptosis and exhaustion and is upregulated in inflammation to reduce immune activation. CTLA4 reduces inflammatory cytokine production in cytotoxic T cells, causing exhaustion and promoting the formation of regulatory T cells. ICIs, especially PD1 and CTLA4 inhibitors, can occasionally activate these immunosuppressive pathways in the liver and may drive unwanted inflammation in this organ [24]. However, it is important to note that ICI-induced hepatitis is observed only in 1–29% of patients, indicating that in most cases, the tightly regulated immune system in the liver is capable of shutting down those unwanted immune responses induced by the ICIs [5]. Therefore, some predisposing factors such as viral infections, obesity, and other liver conditions may be responsible for sensitizing the liver for ICI-hepatitis observed in a small subset of patients. CMV infection in immunocompetent hosts can manifest in liver disorders, including, most commonly, hepatitis [25]. Besides, recent studies have shown that activated CMV-specific memory T cell responses cause hepatitis in patients with combined immune checkpoint inhibitor treatment (nivolumab and ipilimumab) and that treatment with prophylactic valganciclovir an anti-CMV medication, could prevent ICI-related hepatitis [26]. However, we did not observe any correlation between CMV IgG or IgM and ICI-hepatitis in our study. Nevertheless, it is important to note that high CMV-positive IgG titers in our study do not indicate high CMV-specific memory T cell responses in these patients. Besides, it was previously reported that despite remaining globally immunocompetent, CMV-specific T cell memory could be lost in some patients with cancer [27], suggesting possible ineffective immune responses to the CMV infection in advanced melanoma patients. Although we cannot rule out the possible contribution of active CMV infection in these patients, especially during the treatment, predisposing factors other than CMV, such as metabolic conditions, the imbalance between effector and regulatory arms of the immune system, or other viral infections may be responsible for ICI-hepatitis and deserves further investigation to predict ICI-hepatitis.

Immune-related side effects have previously been shown to be associated with better ORR, PFS, and OS in ICI-treated melanoma patients [28]. Accordingly, in our cohort, ICI-hepatitis was significantly associated with tumor disease control response and survival in stage IV melanoma patients. However, a recent Dutch study involving advanced melanoma patients receiving ICIs, majorly ipilimumab, had observed no correlation between hepatitis and survival [13]. Although we are unsure if the difference in the choice of ICI regime may account for such variance in both studies, we would like to warrant further investigation to clarify the findings. Besides, unlike in a previous report [29], we did not observe higher response rates in patients receiving ICI combination therapy compared to PD1 monotherapy in our study. Meanwhile, hepatitis was most frequently observed in patients receiving ICI combination therapy (81%), and in our study, almost 45% of the patients received ICI combination. Hence, it is important to keep in mind that despite statistical adjustment for the ICI regime in our study, a possible influence of combination therapy on tumor responses in patients with hepatitis cannot be completely ruled out.

Interestingly, we observed a strong correlation between high CMV IgG titers and IgM positivity, indicating a possible recent reactivation or primary infection in individuals with high CMV IgG values. The CMV reactivation may generally indicate a weakened immune system or overloaded immune responses [30, 31], suggesting a negative influence on cancer treatment outcomes. In addition, it was previously shown that CMV infection in cancer cells can directly contribute to their growth and treatment resistance [14, 15, 17, 18]. In line with this notion, several reports suggest an association between high CMV IgG levels and increased mortality in advanced cancer patients [32, 33]. Therefore, the negative impact of high CMV IgG values observed in our study may indicate, in general, patients with a highly weakened immune system that is difficult to activate by ICIs. In line with this finding, we also observed reduced monocytes, eosinophils, and neutrophils in the blood of patients with a high CMV IgG ratio. Besides, it is also possible that CMV may have direct effect on melanoma cells and involved in its growth and treatment resistance. Therefore, further studies are required in melanoma models to understand the influence of CMV on ICI resistance in vitro and in vivo. This is particularly important as data linking the relationship between CMV infection and ICI treatment outcomes are limited. In contrast to our findings, there has been a report that high CMV IgG titers in the blood indicate better PFS in a few melanoma patients [34]. However, the findings were based on very few patients in this study and may have contributed to these discrepancies. Nevertheless, evaluating these findings using larger cohorts is strictly necessary, especially since efforts are being made to develop CMV peptides to induce anti-tumor responses in patients [35]. A better understanding of CMV-associated immune responses and their implications for melanoma anti-tumor immune responses may aid in the design of more clinically relevant, tailored, personalized treatment regimes.

While this study aimed to address the relevance of CMV infection to ICI-induced hepatitis and treatment-related outcomes, limitations are associated mainly with the retrospective nature of this study and the availability of biomaterial. Firstly, although it would be exciting in terms of response to therapy, no further analysis of CMV IgM or IgG titers during ICI treatment could be done for all the patients in cohort 1 because of institutional limitations. Secondly, different methods were used for CMV antibody testing in cohorts 1 and 2. Besides, analyzing the peripheral T cell subsets and the IFN-γ-releasing capability of T cells in CMV IgG seropositive patients and in patients with hepatitis could be very informative. Similarly, analyzing the tumor microenvironment for differences in immune cell populations between patients with low and high CMV Ig titers would be interesting. Furthermore, the current study included patients over a time period of nine years; however, it is important to note that the ICI regimes used in this study remain unchanged, and the study focuses on tumor responses and PFS as efficiency outcomes instead of overall survival. However, it is not possible based on retrospective clinical laboratory data. Despite these limitations, to our knowledge, this is the first study to describe the relationship between CMV and hepatitis and ICI outcomes in mCM patients. Our study highlights that CMV may not be related to ICI-hepatitis and that high CMV IgG titers may indicate poor response to the treatment.

## Supplementary Information

Below is the link to the electronic supplementary material.

Supplementary file1 (PDF 299 KB)

## Data Availability

No datasets were generated or analyzed during the current study.
