# Supplementary material for: CMV IgG in the blood is not associated with hepatitis but correlates with poor outcomes in immunotherapy treated melanoma patients
Source: Cancer Immunol Immunother. 2025 Jan 3;74(2):59. doi: 10.1007/s00262-024-03859-3 (PMC11699187; doi:10.1007/s00262-024-03859-3)
Supplement: Supplementary file 1 — Supplementary file1 (PDF 299 KB) [file 262_2024_3859_MOESM1_ESM.pdf]

Supplemental Figure 1

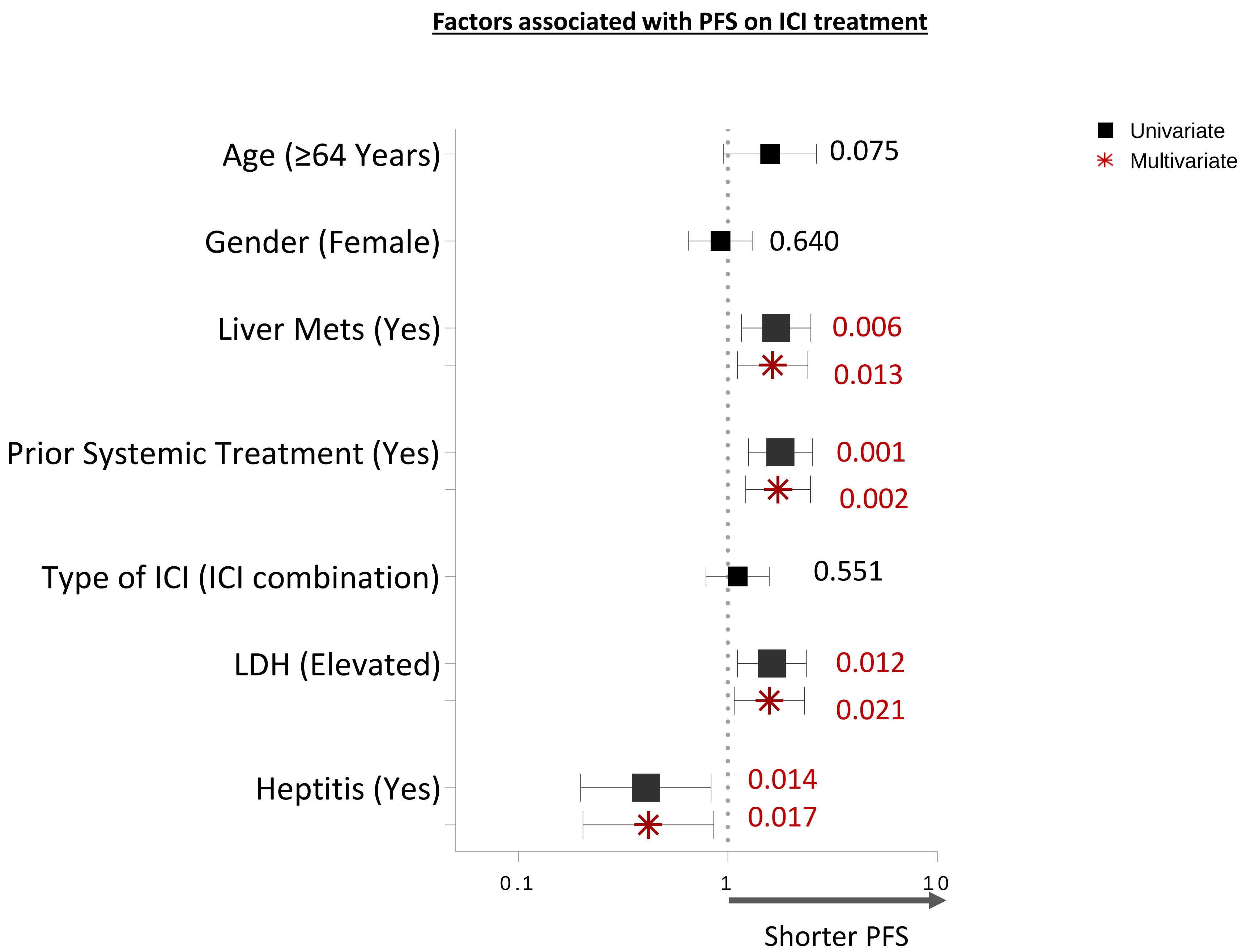

**Supplemental Figure 1:** Forest plot representing the clinical factors associated with PFS in uni (black) and multivariate (red) analysis. The symbols indicate the hazard ratios (HR), and lines indicate the lower and upper 95% CI. p-values are mentioned next to the lines of respective clinical factor. The red colour indicates a significant association of a factor with PFS.

Supplemental Figure 2

(a)

**Factors associated with ICI response on PD1+/-Ipi: combined analysis**

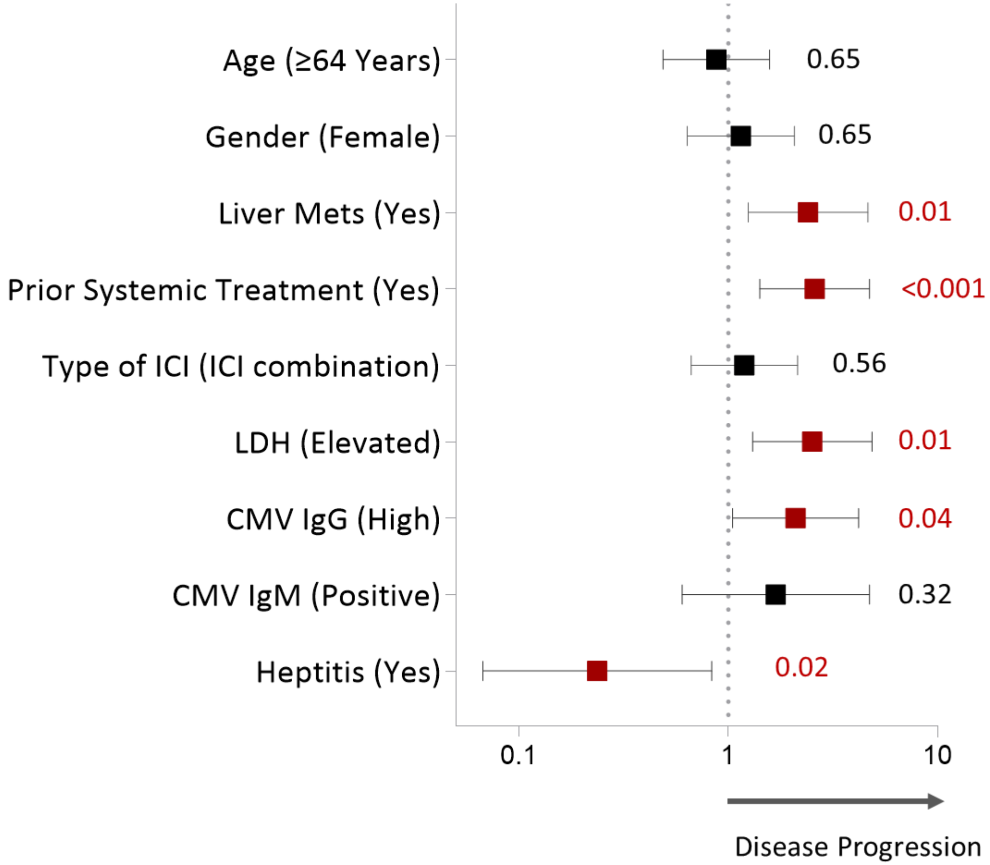


(b)

**CMV IgG, IgM versus disease control response on Ipi+Nivo Combination (Combi) and PD1 Monotherapy (PD1): individual analysis**

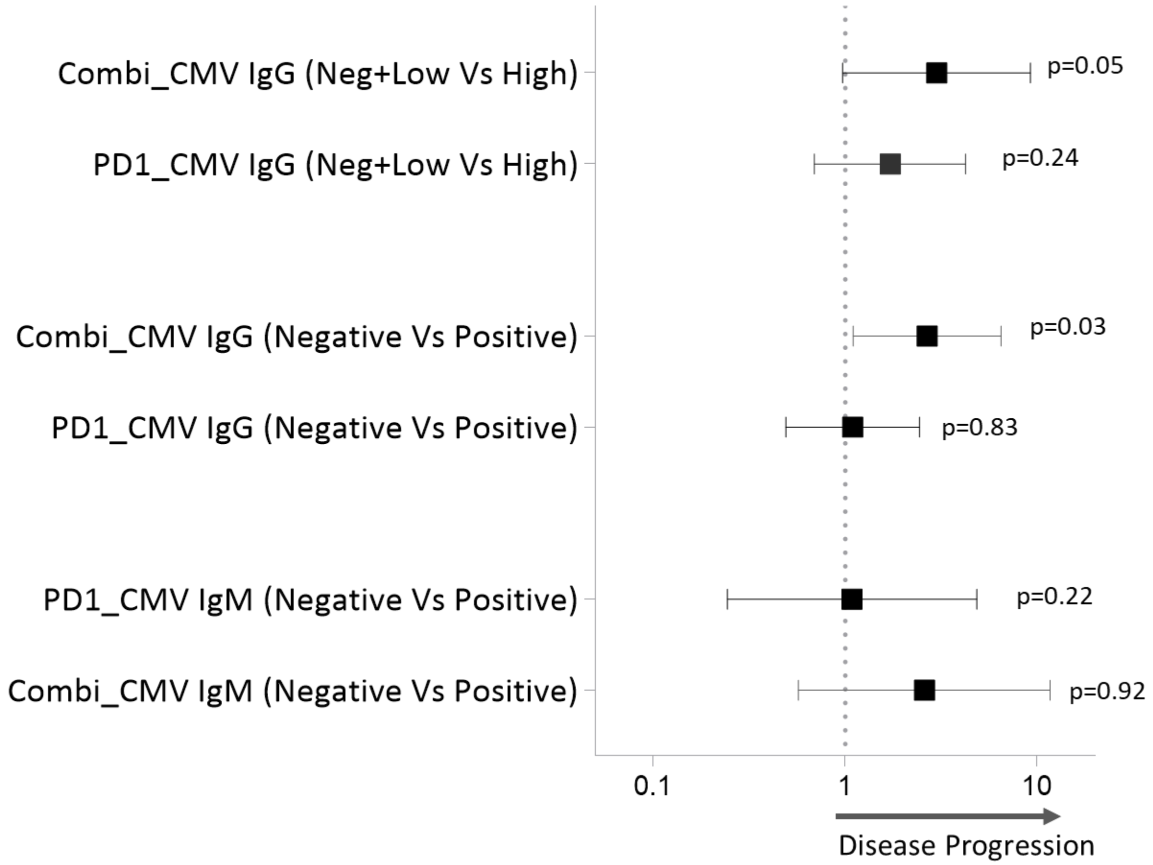

**Supplemental Figure 2:** Forest plots representing the clinical factors associated with disease control response in univariate analysis. The symbols indicate the hazard ratios (HR), and lines indicate the lower and upper 95% CI. p-values are mentioned next to the lines of respective clinical factor. The red colour indicates a significant association of a factor with tumor response.

Supplemental Figure 3

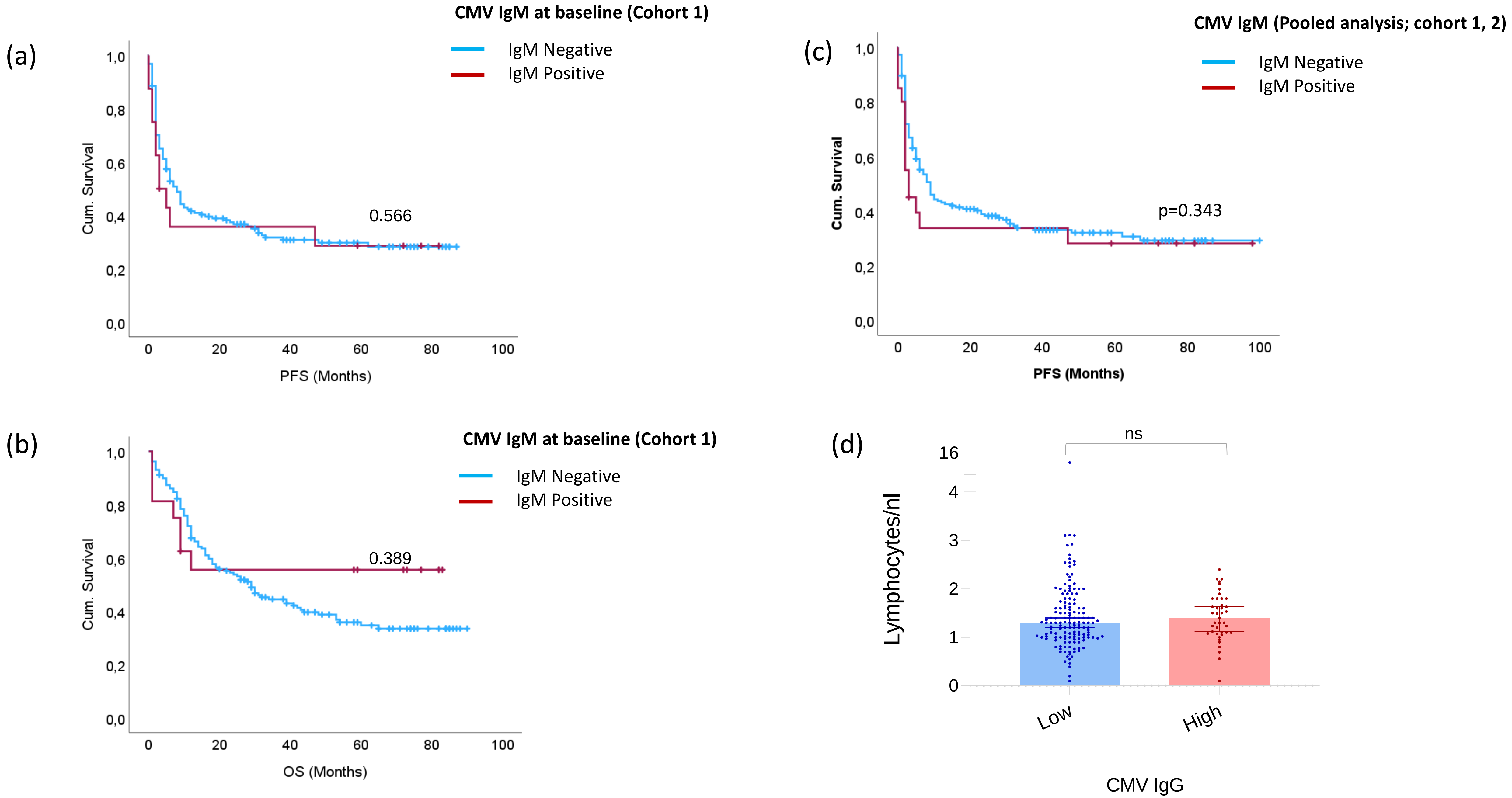

**Supplemental Figure 3.** Kaplan-Meier curves for survival according to CMV IgM negative (blue) or positive (red), (a, c) PFS (b) OS. p-values refer to the log-rank test. (d) Bar charts showing the association between CMV IgG and peripheral absolute lymphocyte counts.

Suppl Table 1

| | Hepatitis (n=7) | No Hepatitis (n= 42) | p-value |
|---|---|---|---|
| **Age in years (range)** | 56 (42-72) | 59 (29-82) | 0.51 |
| **Gender (n (%))** | | | 0.04 |
| Male | 7 (100) | 24 (57) | |
| Female | 0 | 18 (43) | |
| **Tumor Type (n (%))** | | | 0.66 |
| Cutaneous Melanoma | 6 (86) | 30 (71) | |
| Uveal Melanoma | 1 (14) | 5 (12) | |
| Others | 0 | 7 (17) | |
| **Type of ICI (n (%))** | | | 0.05 |
| Pembro | 1 (14) | 24 (57) | |
| Ipi+Nivo | 6 (86) | 18 (43) | |
| **LDH (n (%))** | | | 0.17 |
| Normal | 7 (100) | 30 (71) | |
| Elevated | 0 | 12 (29) | |
| **Other irAEs (n (%))** | | | 0.67 |
| Yes | 3 (43) | 13 (31) | |
| No | 4 (57) | 29 (69) | |
| **Response (n (%))** | | | 0.08 |
| PD | 0 | 17 (40) | |
| DCR | 7 (100) | 25 (60) | |
| **PFS in months (median (95% CI))** | 7 (4-23) | 8 (4-29) | 0.95 |
| **OS in months (median (95% CI))** | 28 (6-73) | 26 (16-42) | 0.77 |
